# Artificial intelligence for predicting paediatric acute kidney injury: a systematic review and meta-analysis

**DOI:** 10.1093/ckj/sfag063

**Published:** 2026-02-28

**Authors:** Rupesh Raina, Parth Shirode, Raghav Shah, Aanya Chepyala, Abhishek Tibrewal, Sidharth Kumar Sethi, Wisit Cheungpasitporn

**Affiliations:** Department of Pediatric Nephrology, Akron Children’s Hospital, Akron, OH, USA; Department of Nephrology, Akron Nephrology Associates/Cleveland Clinic Akron General Medical Center, Akron, OH, USA; Department of Medicine, College of Medicine, Northeast Ohio Medical University, Rootstown, OH, USA; Department of Pediatric Nephrology, Akron Children’s Hospital, Akron, OH, USA; Department of Nephrology, Akron Nephrology Associates/Cleveland Clinic Akron General Medical Center, Akron, OH, USA; Department of Medicine, College of Medicine, Northeast Ohio Medical University, Rootstown, OH, USA; Department of Nephrology, Akron Nephrology Associates/Cleveland Clinic Akron General Medical Center, Akron, OH, USA; Department of Nephrology, Akron Nephrology Associates/Cleveland Clinic Akron General Medical Center, Akron, OH, USA; Pediatric Nephrology, Kidney Institute, Medanta, The Medicity Hospital, Gurgaon, Haryana, India; Division of Nephrology and Hypertension, Mayo Clinic, Rochester, MN, USA

**Keywords:** artificial intelligence, machine learning, paediatric acute kidney injury, paediatric nephrology, predictive modelling

## Abstract

**Background:**

Acute kidney injury (AKI) in hospitalised children is a major complication associated with significant morbidity and mortality. The integration of artificial intelligence (AI)/machine learning (ML) models may enable early detection and risk stratification. This systematic review evaluates the performance of AI/ML models for predicting paediatric AKI across clinical settings.

**Methods:**

We systematically searched PubMed, Embase, and Web of Science for studies applying AI/ML models to predict AKI in paediatric populations. Studies reporting performance metrics such as the area under the curve (AUC), sensitivity, specificity, positive predictive value (PPV), negative predictive value (NPV), accuracy, and F1 score were included.

**Results:**

Among 470 records identified, 11 studies met the inclusion criteria, with 14 AI/ML models used. The overall sample size included 33 949 paediatric patients with an AKI proportion of 12.5%. Meta-analyses of the AUC were conducted on neural network, gradient boosting, and logistic regression. Gradient boosting had the highest pooled AUC of 0.873 (95% confidence interval 0.836–0.909). Random forest demonstrated the highest median sensitivity (0.821), specificity (0.942), PPV (0.860), NPV (0.935), and accuracy (0.821); however, these metrics could not be pooled due to inconsistent reporting and limited validation.

**Conclusion:**

Gradient boosting, random forest, and logistic regression demonstrated reasonable predictive performance for paediatric AKI prediction within specific clinical contexts. However, small sample size, heterogeneity, lack of testing/validation cohorts, insufficient data, and inconsistent patient populations and AKI diagnostic criteria restrict generalisability.

KEY LEARNING POINTS **What was known:**

- Paediatric acute kidney injury (AKI) is associated with high morbidity, mortality, and long-term CKD risk; early detection remains challenging due to the delayed increase in serum creatinine after kidney injury.
- Artificial intelligence (AI)/machine learning (ML) models have shown promise in predicting AKI in adults, but children-specific evidence has been limited.

**This study adds:**

- This systematic review and meta-analysis provide a comprehensive synthesis of currently available AI/ML models developed for predicting paediatric AKI, evaluating their performance across diverse clinical settings.
- Gradient boosting and random forest models showed comparatively strong predictive performance.
- Findings highlight the impact of heterogeneity in AKI definitions, patient cohorts, and feature selection on model performance.

**Potential impact:**

- This study supports the development of standardized, multicentre paediatric datasets to improve AI/ML model reliability.
- It encourages integration of interpretable AI models into electronic health records for real-time paediatric AKI risk stratification.
- It may inform clinical decision support tools that enable earlier interventions, potentially improving paediatric AKI outcomes.

## INTRODUCTION

Acute kidney injury (AKI) is a major complication occurring in hospitalised children [1], causing prolonged hospital stays and higher mortality in critically ill children in paediatric intensive care units (PICUs) [2, 3]. A multinational prospective study by Kaddourah *et al*. [3] reported a progressive increase in 28-day mortality associated with the severity of paediatric AKI. AKI also increases the long-term risk of chronic kidney disease (CKD) in children admitted to the PICU [4]. In a prospective cohort study by Mammen *et al*. [4], 10.3% of children developed CKD 1–3 years after AKI incidence, with 46.8% at risk for CKD. Additionally, even mild or moderate AKI places children at a higher risk of long-term complications such as hypertension, proteinuria, and CKD in adulthood [4, 5]. Thus, earlier identification of children at high risk of AKI enables prompt and targeted interventions. AKI definitions such as paediatric RIFLE (pRIFLE; risk, injury, failure, loss of kidney function, end-stage renal disease) criteria, AKI Network (AKIN) criteria, and, most recently, the Kidney Disease: Improving Global Outcomes (KDIGO) classification system are used to diagnose paediatric AKI. However, these criteria are based on serum creatinine and urine output, which are suboptimal, as serum creatinine reflects changes in glomerular filtration rate (GFR) and may take 24–36 hours to increase after a significant renal injury, potentially delaying diagnosis and timely care [6].

The use of artificial intelligence (AI) as a method of AKI detection and risk stratification via clinical decision tools has become increasingly common [7]. AI leverages machine learning (ML) and problem-solving for AKI diagnosis by identifying patterns, relationships, and inputs from given data and then applying these externally to novel data [8]. ML, an AI subset, applies data-driven algorithms such as linear and logistic regression, random forests, gradient boosting, XGBoost, and deep neural networks to predict AKI outcomes [8]. Each type of AI model has varying levels of efficacy, depending on the specific algorithms utilised and the quality of input data [9–11]. We aimed to systematically review and compare statistical analyses and measures of model performance of existing AI/ML models for predicting paediatric AKI and to identify the specific outcomes associated with each model type.

## MATERIALS AND METHODS

### Study design

Our study was designed according to the Preferred Reporting Items for Systematic Review and Meta-analysis Protocols (PRISMA-P) guidelines and registered on PROSPERO (CRD42024570403).

### Data sources and search strategy

A comprehensive search was conducted across PubMed, Embase, and Web of Science to identify studies utilising AI and ML models for predicting AKI. Search terms included keywords associated with AI, ML, and AKI in children. A comprehensive search strategy can be found in Supplementary Method 1.

### Eligibility criteria

Studies were included if they reported key performance metrics such as area under the curve (AUC), sensitivity, specificity, positive predictive value (PPV), negative predictive value (NPV), accuracy, and F1 score. Additionally, only cross-sectional, prospective, randomised controlled trials (RCTs) and retrospective studies published until July 2024 were included. A detailed PICOS (population, intervention, comparator, outcome, study design) table can be found in Supplementary Method 2.

### Study selection

After removing duplicate studies using reference management software, two independent reviewers (A.C. and R.S.) conducted a two-stage screening process. First, titles and abstracts were reviewed to exclude irrelevant studies, followed by a full-text assessment based on predefined inclusion and exclusion criteria, ensuring the selection of studies focused on paediatric AKI prediction using AI/ML models with reported performance metrics. Any disagreements were resolved by a third independent reviewer (R.R).

### Risk of bias assessment

Two independent reviewers (A.C. and R.S.) graded each study for its level of evidence based on methodological quality, validity, and applicability using the Newcastle–Ottawa Scale (NOS) for quality assessment (Supplementary Table 1 and Supplementary Fig. 1). Any disagreements were resolved by a third reviewer (R.R).

### Data extraction and statistical analysis

The AI performance metrics and their 95% confidence intervals (CIs) were extracted for each of the included studies. The degree of between-study heterogeneity was assessed using the *I*^2^ test, where *I*^2^ ≥ 50% indicated high heterogeneity. The overall (pooled) estimate was calculated with a random effects model for high heterogeneity and a fixed effects model for low heterogeneity. A forest plot was used to visualise these outcomes in each study and the combined estimated outcomes with their 95% CIs. Publication bias was assessed with Egger’s test. DeLong’s test was used to compare the ROC curves of the two models. To identify the source of heterogeneity, subgroup analyses were conducted based on different variables (study design, age of the subjects, data source, AKI definition). The data for some of the outcomes have been reported as median [interquartile range (IQR)] (due to the inability to conduct the meta-analysis). A *P*-value ≤.05 was set as the level of significance. All statistical analyses were performed with R version 3.1.0 (R Foundation for Statistical Computing, Vienna, Austria).

## RESULTS

### Included studies

**Figure 1:**
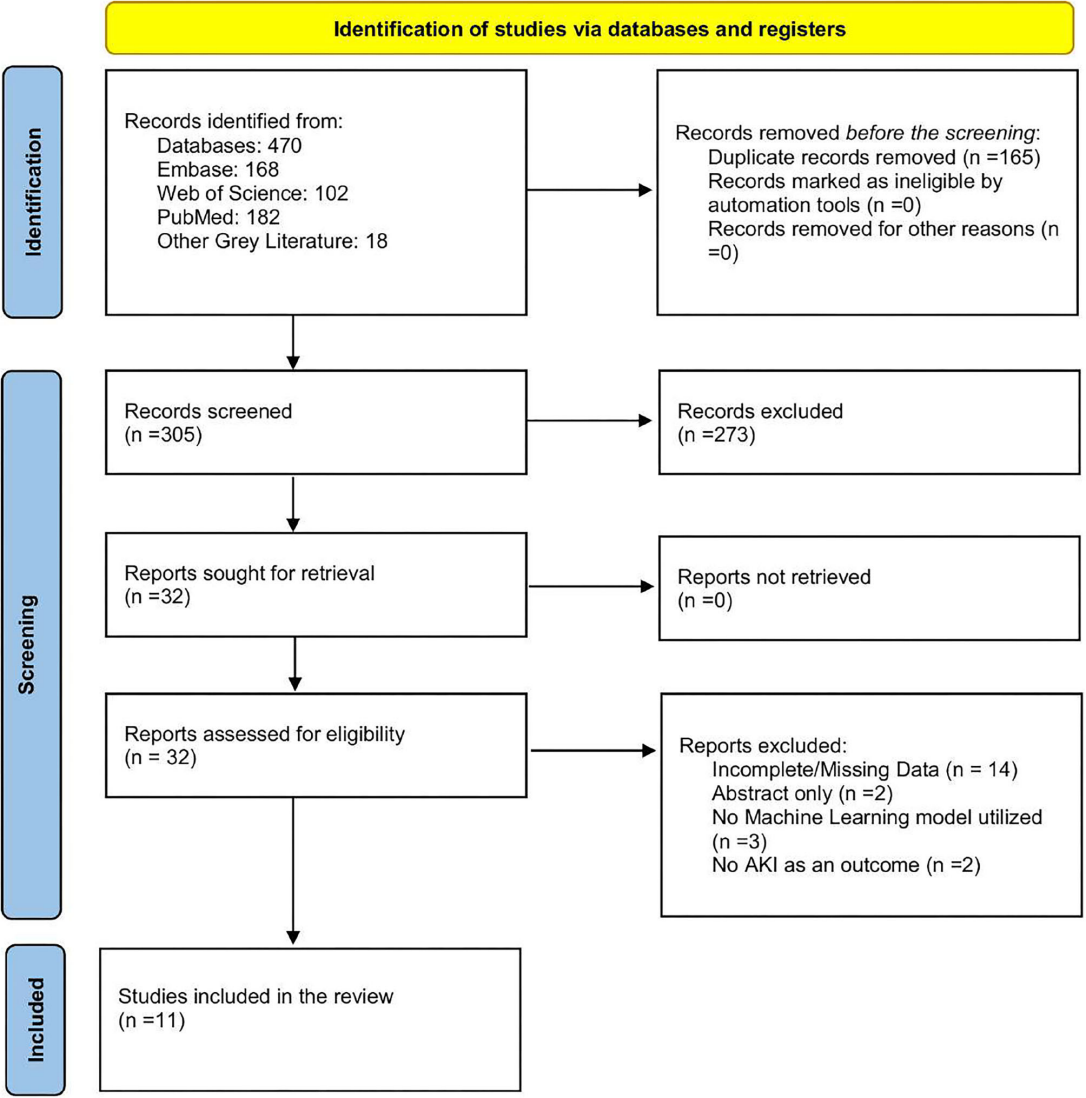
PRISMA (Preferred Reporting Items for Systematic reviews and Meta-Analyses) flow diagram.

We included 11 studies involving children with AKI out of 470 records from our initial search (Fig. 1). The overall sample size across these studies was 33,949 (ranging from 19 to 16,863 across various studies) and the proportion of patients with AKI was 12.5% (4260/33 949) (ranging from 8.9 to 83.1% across various studies). The mean or median age reported across studies was 1 year, with study-level mean/median values ranging from 0.17 to 8.27 years. AKI was defined based on KDIGO guidelines (*n* = 8), pRIFLE criteria (*n* = 2) and both KDIGO and pRIFLE (*n* = 1). Of the 11 included studies, 5 included children undergoing cardiac surgery or admitted to the paediatric cardiac intensive care unit [11–15], 2 included critically ill children [16, 17], 2 included first allogeneic haematopoietic stem cell transplantation (HSCT) [18, 19], 1 included congenital heart surgery [9] and 1 included hospitalised children with at least two creatinine values [20]. There were nine retrospective studies and two prospective cohort studies. Most of the studies were conducted in China (*n* = 4), followed by the USA (*n* = 2), Poland (*n* = 2), UK (*n* = 1), Italy (*n* = 1), and 1 study was conducted in both the USA and UK. Supplementary Table 2 describes the characteristics of all included studies.

### Characteristics of AI/ML models in the included studies

A total of 14 different AI/ML models were reported in these studies. Additionally, the studies categorised cohorts into training/derivation, testing, and validation (internal/external) groups, each of which was analysed as a separate model in our study. The AI models included logistic regression, random forest, gradient boosting, support vector machine, neural network, ensemble learning technique, decision tree, extra tree, genetic algorithm, clinical, top AUC, GaussianNB, dipole, and KNN (Supplementary Table 3). The number of features ranged from 3 to 83 across the models. The features considered for the models across these studies comprised demographic, vitals, fluid intake, blood gas analysis, laboratory parameters, therapies, surgery, and operative characteristics and biomarkers [kidney injury molecule 1 (KIM-1), interleukin-18 (IL-18), and neutrophil gelatinase-associated lipocalin (NGAL)]. Ten studies included preoperative features, six studies included demographic features, three studies included intraoperative features, and three studies included postoperative features (some studies had more than one feature type). Biomarkers (KIM-1, IL-18, and NGAL) were considered in one study (training cohort with no validation or testing cohort). The training cohort mostly used demographic features, preoperative features and intra-operative features, while the validation cohort used demographic features and preoperative features, whereas the testing cohort used demographic features, preoperative features, intra-operative features, and postoperative features. The feature selection method for these AI models included Shapley additive explanations [SHAP; three studies: two retrospective and one prospective; two studies used electronic health records (EHRs) data), least absolute shrinkage and selection operator (LASSO; two studies; both retrospective), feature importance (one study; retrospective; EHRs data source), recursive feature selection (one study; prospective), forward selection (one study; retrospective), random forest variable importance plot and multicollinearity testing (one study; retrospective) and a combination of four methods (LASSO, Boruta algorithm, random forest recursive feature elimination and random forest filtering; one study; retrospective; EHRs data source), with the feature selection method not reported in two studies. The most important features identified by these AI models differed across the cohorts, as shown in Supplementary Table 4.

### Area under the curve (AUC)

**Figure 2:**
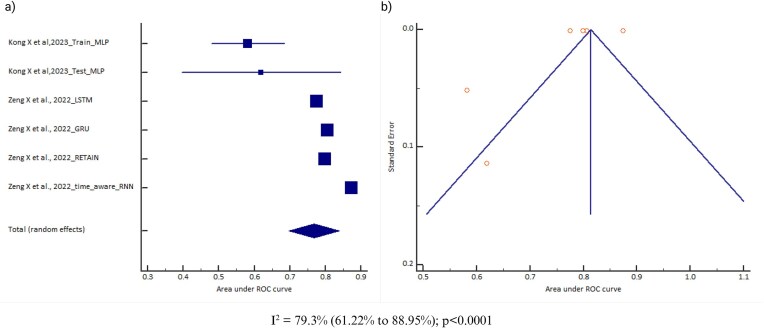
**(a)** Forest plot of the meta-analysis of neural network model AUC across different studies. The lower diamond in the graph represents the pooled estimate. **(b)** Funnel plot of the meta-analysis of neural network model AUC across different studies. *I*^2^: heterogeneity; p: probability value.

**Figure 3:**
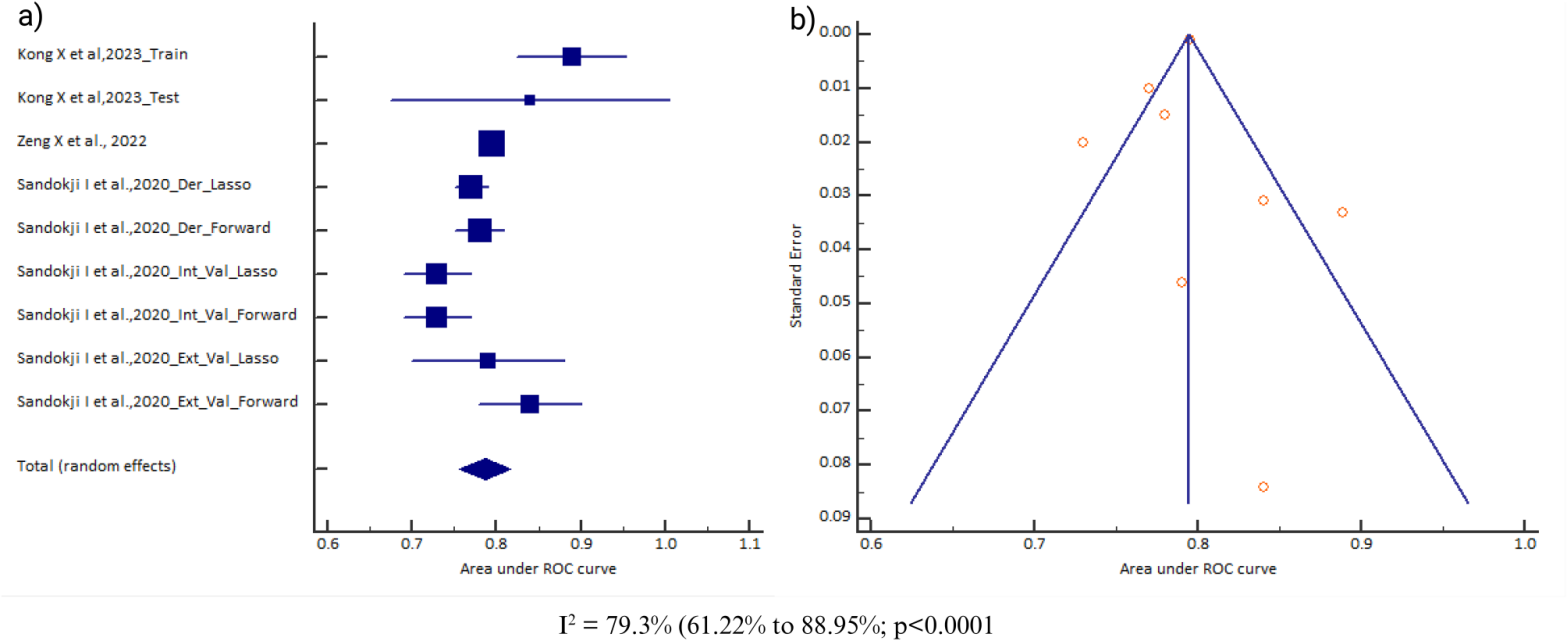
**(a)** Forest plot of the meta-analysis of the logistic regression model AUC across different studies. The lower diamond in the graph represents the pooled estimate. **(b)** Funnel plot of the meta-analysis of the logistic regression model AUC across different studies. *I*^2^: heterogeneity; p: probability value.

**Figure 4:**
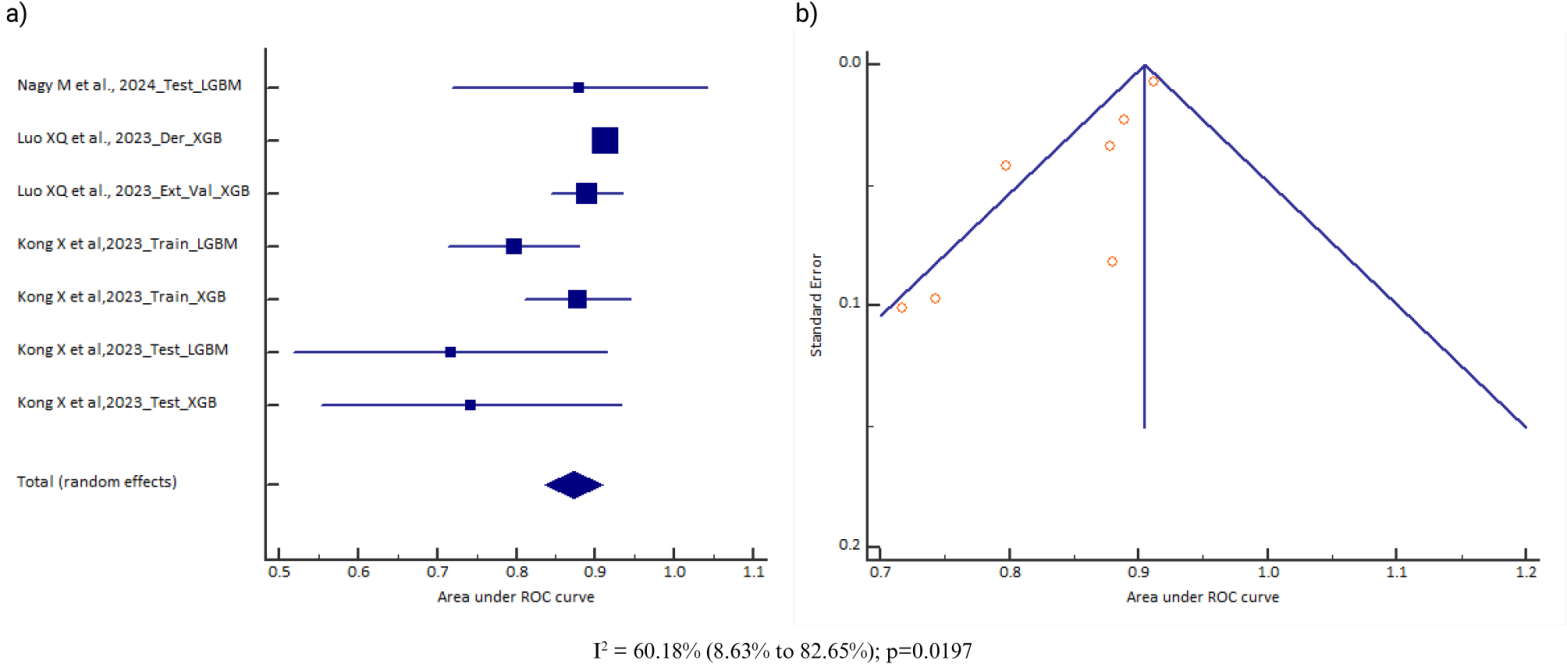
**(a)** Forest plot of the meta-analysis of the gradient boosting model AUC across different studies. The lower diamond in the graph represents the pooled estimate. **(b)** Funnel plot of the meta-analysis of the gradient boosting model AUC across different studies. *I*^2^: heterogeneity; p: probability value.

All 14 AI models included data on the AUC. However, data for eight AI model types were reported from only one study and were therefore excluded from the analysis. Also, models for which the 95% CI of the AUC was not reported were not considered (due to a lack of standard error required for the meta-analysis). There were two cohorts for random forest, three cohorts for support vector machine, six cohorts for neural network, seven cohorts for gradient boosting, and nine cohorts for logistic regression. A meta-analysis of the AUC was conducted for three models (neural network, gradient boosting, and logistic regression), but was not done for random forest and support vector machine (due to fewer than five cohorts for a meaningful output). The pooled AUC of the neural network model was 0.768 (95% CI 0.696–0.839) [*I*^2^ = 99.91% (range 99.89–99.92), *P* < .0001, random effects, six cohorts, *n* = 13 678; Table 1a]; for the logistic regression model it was 0.786 (95% CI 0.756–0.817) [*I*^2^ = 79.3% (range 61.22–88.95), *P* < .0001, random effects, nine cohorts, *n* = 20 466; Table 1b] and for gradient boosting model it was 0.873 (95% CI 0.836–0.909) [*I*^2^ = 60.18% (range 8.63–82.65), *P* = .0197, random effects, seven cohorts, *n* = 4212; Table 1c]. However, the pooled AUC was not observed to be significantly different across these three models (Delong’s test *P* = .0634; *P* = .1872 and *P* = .8153). The observed heterogeneity might be due to differences in the study populations, data source, and AKI definition, which can influence the pooled effect sizes and CIs. There was no evidence of publication bias for the logistic regression model (Egger’s test *P* = .4720) and neural network model (Egger’s test *P* = .9117), but there was for gradient boosting (Egger’s test *P* = .009). A significant Egger’s test and asymmetry funnel plot for gradient boosting suggest publication bias and a possible inflation of the reported AUC; therefore, a more cautious interpretation would be advisable. Figs. 2–4 provide the forest plots and funnel plots for these three models across different studies.

**Table 1:** Meta-analysis of the AUC for three AI/ML models in assessing AKI among pediatric patients.

Study/cohort/model	AKI/model sample size, *n*	AUC (95% CI)	Random weight (%)
Neural network			
Kong X *et al*., 2023_Train_MLP	54/108	0.582 (0.480–0.684)	14.1
Kong X *et al*., 2023_Test_MLP	13/26	0.619 (0.396–0.842)	6.73
Zeng X *et al*., 2022_LSTM	331/3386	0.775 (0.773–0.777)	19.79
Zeng X *et al*., 2022_GRU	331/3386	0.806 (0.804–0.808)	19.79
Zeng X *et al*., 2022_RETAIN	331/3386	0.799 (0.797–0.801)	19.79
Zeng X *et al*., 2022_time_aware_RNN	331/3386	0.874 (0.872–0.876)	19.79
Pooled result (random effects)	1391/13 678	0.768 (0.696–0.839)	100
Logistic regression			
Kong X *et al*., 2023_Train	54/108	0.889 (0.824–0.954)	9.31
Kong X *et al*., 2023_Test	13/26	0.840 (0.675–1.000)	2.77
Zeng X *et al*., 2022	331/3386	0.795 (0.793–0.797)	16.38
Sandokji I *et al*., 2020_Der_Lasso	516/5072	0.77 (0.750–0.790)	15.32
Sandokji I *et al*., 2020_Der_Forward	516/5072	0.78 (0.751–0.809)	14.17
Sandokji I *et al*., 2020_Int_Val_Lasso	207/2299	0.73 (0.691–0.769)	12.81
Sandokji I *et al*., 2020_Int_Val_Forward	207/2299	0.73 (0.691–0.769)	12.81
Sandokji I *et al*., 2020_Ext_Val_Lasso	27/1102	0.79 (0.700–0.880)	6.62
Sandokji I *et al*., 2020_Ext_Val_Forward	27/1102	0.84 (0.779–0.901)	9.81
Pooled result (random effects)	1898/20 466	0.786 (0.756–0.817)	100
Gradient boosting			
Nagy M *et al*., 2024_Test_LGBM	30/81	0.880 (0.719–1.000)	4.56
Luo XQ *et al*., 2023_Der_XGB	564/3278	0.912 (0.898–0.926)	35.46
Luo XQ *et al*., 2023_Ext_Val_XGB	51/585	0.889 (0.844–0.934)	23.84
Kong X *et al*., 2023_Train_LGBM	54/108	0.797 (0.715–0.879)	12.93
Kong X *et al*., 2023_Train_XGB	54/108	0.878 (0.811–0.945)	16.69
Kong X *et al*., 2023_Test_LGBM	13/26	0.717 (0.519–0.915)	3.14
Kong X *et al*., 2023_Test_XGB	13/26	0.743 (0.553 to 0.933)	3.38
Pooled result (random effects)	779/4212	0.873 (0.836 to 0.909)	100

Neural network included ANN, KNN, Multilayer Perceptron (MLP), LSTM, gate recurrent units, RETAIN and time-aware attention-based RNN. Logistic regression included logistic regression, forward selection method, LASSO. Gradient boosting included GBM, light GBM and XG Boost.

Train: training; Test: testing; MLP: multilayer perceptron; LSTM: long short-term memory; GRU: gated recurrent unit; RETAIN: Reverse Time Attention model; Time_aware_RNN: time-aware attention-based recurrent neural network; Der: derivation; Int_Val: internal validation; Ext_Val: external validation; LGBM: Light Gradient Boosting Machine; XGB: Extreme Gradient Boosting.

To investigate the source of heterogeneity, subgroup analysis was conducted based on different variables (study design, age of the subjects, data source, and AKI definition) as shown in the Supplementary Table 5. The study design was a retrospective cohort study for all the studies included in each of the AI models. The pooled AUC for the AI models was observed to differ based on the stratification of the variables. For the data source variable, the pooled AUC was observed to be higher for gradient boosting [0.89 (95% CI 0.88–0.91)] using an EHR data source and for logistic regression [0.84 (95% CI 0.77–0.92)] using a non-EHR data source. For the age of the subject variable, the pooled AUC was observed to be higher for gradient boosting [0.90 (95% CI 0.88–0.92)] among ≥1 year and for logistic regression [0.87 (95% CI 0.82–0.91)] among <1 year. For the AKI definition variable, the pooled AUC was observed to be higher for gradient boosting [0.89 (95% CI 0.88–0.91)] using the KDIGO guideline and for logistic regression [0.87 (95% CI 0.82–0.91)] using KDIGO and RIFLE criteria. However, for variables where logistic regression is observed to perform better than gradient boosting, the 95% CI AUCs are overlapping for the two models, indicating that even gradient boosting can be equally effective for those variables. Also, the subgroup analyses were based on a limited number of cohorts, so the findings should be interpreted with caution.

### Sensitivity/recall, specificity, PPV, NPV, accuracy, and F1 score

The data for sensitivity were reported in 10 studies, specificity in 6 studies, PPV in 9 studies, NPV in 6 studies, accuracy in 7 studies, and F1 score in 3 studies. However, the 95% CI was reported in these studies for only seven cohorts for sensitivity (four for neural network, one for gradient boosting, one for logistic regression and one for support vector machine), for only seven cohorts for accuracy (four for neural network, one for gradient boosting, one for logistic regression and one for support vector machine), for no cohorts for specificity, for one cohort for PPV (one for gradient boosting), for no cohorts for NPV and for one cohort for F1 score (one for gradient boosting). Due to a lack of standard error (missing in the majority of studies, making it unfeasible to conduct imputation or modelling method), no meta-analysis was conducted for all these performance metrics. Therefore, the data for these outcomes have been reported as median (IQR). Also, detailed performance metrics for different AI models across the included studies have been reported in Supplementary Table 6.

The median of sensitivity was observed to be highest for the random forest model [0.821 (IQR 0.778–0.850)] and lowest for the ensemble learning technique [0.553 (IQR 0.411–0.694)], as shown in Supplementary Table 7a. The median of specificity was observed to be highest for the random forest model [0.942 (IQR 0.923–0.961)] and lowest for the neural network [0.767 (IQR 0.739–0.824)], as shown in Supplementary Table 7b. The median of PPV was observed to be highest for the random forest model [0.860 (IQR 0.833–0.880)] and lowest for the support vector machine [0.584 (IQR 0.549–0.650)], as shown in Supplementary Table 7c. The median of NPV was observed to be highest for the random forest model [0.935 (IQR 0.928–0.943)] and lowest for the neural network [0.660 (IQR 0.648–0.768)], as shown in Supplementary Table 7d. The median of accuracy was observed to be highest for the random forest model [0.821 (IQR 0.765–0.856)] and lowest for support vector machines [0.630 (IQR 0.477–0.766)], as shown in Supplementary Table 7e. The median of F1 score was observed to be highest for gradient boosting [0.788 (IQR 0.702–0.794)] and lowest for neural networks [0.675 (IQR 0.668–0.683)], as shown in Supplementary Table 7f. The data for random forest (for each of the outcomes) is reported from only one cohort from each of the studies; the method for handling overfitting is not clearly described in these studies.

## DISCUSSION

To our knowledge, this is the first systematic review and meta-analysis to evaluate the effectiveness of various AI models in predicting AKI in children. Traditional scoring systems and regression models have limitations, highlighting the need for advanced ML-based predictive models to enhance accuracy, interventions, and resource allocation [21].

The meta-analysis of the AUC was done on only three AI models: neural network, logistic regression, and gradient boosting. Gradient boosting had the highest pooled AUC of 0.873 (95% CI 0.836–0.909), followed by logistic regression and neural network. None of the three AI models showed a clear performance advantage over the others in terms of AUC. Gradient boosting achieved the highest median F1 score of 0.788 (IQR 0.702–0.794), a metric that integrates precision and recall to assess overall model accuracy. This suggests that ensemble methods like gradient boosting could be effective in clinical settings where missing true positive AKI cases or overburdening with false alerts are crucial.

In our study, random forest models showed the highest median sensitivity, specificity, PPV, NPV, and overall accuracy. These findings suggest that random forest models may perform well in accurately identifying true paediatric AKI cases while reducing false positives. When compared with other models, the logistic regression model could allow clinicians to understand which factors have the most significant impact when predicting AKI in children. In our study, logistic regression showed moderate results in the AUC and other variables, producing stable performance across diverse patient cohorts. However, this model may underfit complex paediatric AKI datasets unless extended methods like LASSO or forward selection are used [11, 20]. Although the neural network model achieved a high AUC in the meta-analysis, its other results indicate it can misclassify a relatively high proportion of AKI cases. This trend suggests their less robust predictive capacity for the paediatric AKI dataset included in our study. However, these deep-learning models often require large, well-harmonised datasets to learn subtle patterns [22]. The lack of uniform study designs can compromise their performance.

Importantly, differences in reported performance across model types should be interpreted in the context of the strength of available evidence. Neural network, logistic regression, and gradient boosting models were the only approaches with sufficient cohorts and reported CIs to permit meta-analysis of the AUC, providing more reliable pooled estimates of model performance. In contrast, although random forest models demonstrated higher median sensitivity, specificity, and predictive values, these metrics could not be pooled due to inconsistent reporting and lack of standard errors and therefore represent descriptive, hypothesis-generating findings rather than definitive evidence of superiority. As a result, apparent differences between models reflect variation in reporting practices and study design as much as true algorithmic performance.

The findings from adult studies bring a comparative perspective, highlighting the variability of AI/ML models in predicting AKI across diverse clinical settings. A review by Bacci *et al*. [23] studying an adult cohort showed a similar trend where the performance of the deep learning model (AUC 0.907 in critical care AKI; AUC 0.797 in hospitalised patients AKI) was similar to logistic regression (AUC 0.877 in critical care AKI; AUC 0.789 in hospitalised patients) for two different clinical settings and patient populations. Raina *et al*. [24] reported high AUCs of 0.852 for the AI/ML models Broad Learning System and Elastic Net Final, which showed that model adaptability and feature selection are important factors in optimizing their performance. Adult cohorts frequently add more complex features, including higher burdens of comorbidities and wider aetiological spectra of AKI, which reduce the direct generalisability of findings to paediatric populations.

Our study has major limitations, including substantial heterogeneity in patient populations, AKI definitions (KDIGO versus pRIFLE), and clinical settings, which restrict the generalizability of the results. The included cohorts spanned infancy through adolescence and encompassed diverse contexts such as cardiac surgery ICUs, general PICUs, HSCT units, and general wards—each with distinct baseline renal physiology, creatinine kinetics, and AKI risk profiles. These differences are particularly pronounced in infants, in whom small absolute creatinine changes may be magnified, and baseline values are often uncertain, complicating AKI classification. AKI was defined using KDIGO, pRIFLE, or both, each employing different creatinine and urine output thresholds and time windows, increasing the likelihood of outcome misclassification across studies. Together, this heterogeneity contributed to the high between-study *I*^2^ values and limits strict cross-study comparability; consequently, the pooled AUCs should be interpreted as summary averages across heterogeneous paediatric contexts rather than precise estimates for any specific age group or AKI definition. Additionally, several models lacked external validation, raising concerns for overfitting and limiting real-world applicability. Given these constraints, findings from studies without validation cohorts were included but must be interpreted cautiously.

Standardised data collection across centres, including consistent AKI definitions, systematic inclusion of relevant biomarkers, and comprehensive feature engineering, could enhance the effectiveness of these models. For example, Wainstein *et al*. [25] found that out of hundreds of AKI prediction studies, only three met strict criteria for external validation in general hospital patients. Future studies are required to further develop and refine ML models in paediatric-specific contexts, taking into consideration age-specific variables like serum creatinine, total body fluid, body surface area, developmental physiology, and genetic predispositions. Moreover, AI model integration into EHRs to provide real-time risk stratification and decision support would revolutionize AKI management in many settings, especially those with a high patient turnover, such as ICUs. This could be achieved by collaborative efforts of paediatric nephrologists, data scientists, and software developers. It should prioritize the development of interpretable AI models with clear decision pathways, standardised application programming interfaces, and data formats across different healthcare systems for seamless system integration and provide comprehensive training programs incorporating both technical and clinical perspectives. Success in these areas will be critical for the widespread adoption and optimal utilisation of AI/ML models in paediatric AKI prediction and management.

Taken together, the central finding of this review is not that one AI model is universally superior, but rather that multiple modelling approaches—including gradient boosting, random forest and logistic regression—can achieve reasonable discrimination for paediatric AKI prediction when developed within specific clinical contexts. However, this systematic review revealed substantial between-study heterogeneity, which significantly limits the generalisability of these findings across heterogeneous paediatric populations. Apparent differences in reported performance across models appear to be driven less by algorithm choice alone and more by cohort characteristics, feature selection, outcome definitions and validation strategies. Accordingly, it remains uncertain whether the observed performance metrics reflect the intrinsic predictive capability of the models or are influenced by methodological factors such as study design, sample size, outcome misclassification and lack of external validation. Standardised data collection, incorporation of age-specific biomarkers, transparent reporting of performance metrics and rigorous multicentre external validation across diverse paediatric settings are therefore essential to improve model reliability. Future research should prioritise large-scale validation studies and the integration of interpretable AI models into EHRs to support real-time risk stratification and clinical decision-making. By addressing these gaps, AI-driven predictive models may evolve from proof-of-concept tools into clinically reliable systems capable of enabling timely interventions and improving outcomes in paediatric AKI.

## Supplementary Material

sfag063_Supplemental_Files

## Data Availability

All data generated or analysed during this study are included in this published article and its supplementary information files.
