# Supplementary material for: Artificial intelligence for predicting paediatric acute kidney injury: a systematic review and meta-analysis
Source: Clin Kidney J. 2026 Feb 28;19(5):sfag063. doi: 10.1093/ckj/sfag063 (PMC13134450; doi:10.1093/ckj/sfag063)
Supplement: sfag063_Supplemental_Files [file sfag063_supplemental_files.zip › Supplementary Document AI AKI.docx]

**Supplementary Document**

**Title: Artificial Intelligence for Predicting Pediatric Acute Kidney Injury: A Systematic Review and Meta-Analysis**

**Corresponding author:**

Rupesh Raina, MD, FAAP, FACP, FASN, FNKF.

Department of Nephrology, Akron Children’s Hospital,

214 West Bowery Street, Akron, OH 44308, USA;

Department of Nephrology, Akron Nephrology Associates/Cleveland Clinic Akron General Medical Center, Akron, OH, USA.

Email: [rraina@akronchildrens.org](mailto:rraina@akronchildrens.org).

**Content:**

**Supplemental Method 1 ………………………………………………………….. 2**

**Supplemental Method 2 ………………………………………………………….. 3**

**Supplemental Table 1 ………………..…………………………………………… 4**

**Supplemental Figure 1………………..…………………………………………….5**

**Supplemental Table 2 ………………..……………………………………………..6**

**Supplemental Table 3 ………………..……………………………………………..7**

**Supplemental Table 4 ………………..……………………………………………..8**

**Supplemental Table 5 ………………..……………………………………………..12**

**Supplemental Table 6 ………………..……………………………………………..14**

**Supplemental Table 7 ………………..……………………………………………..17**

**Supplemental Method 1:** Comprehensive search strategy

The PubMed search strategy was developed using a combination of free-text keywords and Medical Subject Headings (MeSH). Free-text terms were used as the primary approach to maximize sensitivity and capture recently indexed or not-yet-indexed articles, while MeSH terms were incorporated to enhance indexing precision. The search was conducted from database inception through July 2024.

**Pubmed –182**

("Acute Kidney Injury"[MeSH Terms] OR "Acute Kidney Injury"[All Fields] OR "Acute Renal Failure"[All Fields] OR "Renal Injury"[All Fields] OR "Renal Failure"[All Fields]) AND "English"[Language] AND ("Artificial Intelligence"[MeSH Terms] OR "Artificial Intelligence"[All Fields] OR "Machine Learning"[All Fields] OR "Deep Learning"[All Fields] OR "Neural Network"[All Fields]) AND ("Child"[MeSH Terms] OR "Infant"[MeSH Terms] OR "Pediatrics"[MeSH Terms] OR "Pediatrics"[All Fields] OR "Children"[All Fields] OR "Adolescents"[All Fields] OR "Neonates"[All Fields] OR "Infants"[All Fields] OR "Kids"[All Fields]) AND English[lang]

**Web of Science –102**

ALL=("Acute Kidney Injury" OR "Acute Renal Failure" OR "Renal Injury" OR "Renal Insufficiency") AND ALL=("Artificial Intelligence" OR "Machine Learning" OR "Deep Learning" OR "Expert System" OR "Cognitive Computing" OR "Neural Network") AND ALL=("Pediatrics" OR "Children" OR "Neonates" OR "Infants" OR "Adolescents") AND Language=(English)

**Embase- 168**

('Acute Kidney Injury' OR 'Renal Injury' OR 'Renal Insufficiency') AND ('Artificial Intelligence' OR 'Machine Learning' OR 'Deep Learning' OR 'Neural Network' OR 'Cognitive Computing') AND ('Pediatrics' OR 'Children' OR 'Adolescents' OR 'Neonates' OR 'Infants') AND [english]/lim

**Supplemental Method 2:** PICOS criteria

**Population:**
Include studies with more than one patient hospitalized in ICU, post-operative, or any other setting, aged 18 years or younger without a diagnosis of AKI at admission.
Exclude patients older than 18 years or with a diagnosis of AKI at admission.

**Intervention:**
Include studies using Artificial Intelligence, Machine Learning, Deep Learning, logistic regression, broad learning systems, extreme gradient boost, random forest models, or support vector machines.
Exclude studies where none of the above techniques are applied to patient profiles.

**Comparison:**
Include studies comparing different Artificial Intelligence, Machine Learning, or Deep Learning models.
Exclude studies that do not compare or list other such models.

**Outcomes:**
Include studies reporting AKI incidence, AKI staging, model Area Under the Curve (AUC), accuracy, sensitivity, specificity, negative predictive value, or positive predictive value.
Exclude studies with no follow-up, non-efficacy outcomes, or without AKI outcome prediction.

**Study Types:**
Include cross-sectional studies, prospective and retrospective studies, randomized clinical trials, controlled studies, observational studies, and case reports.
Exclude systematic reviews, literature reviews, abstracts, or duplicate case reports.

​​**Supplemental Table 1:** Newcastle Ottawa Risk of Bias Quality Assessment

|  | **Selection** | | | | **Comparability** | **Exposure** | | |  |
| --- | --- | --- | --- | --- | --- | --- | --- | --- | --- |
| **Author** | **Representativeness of exposed cohort** | **Selection of the non-exposed cohort** | **Ascertainment of exposure** | **Demonstration that outcome of interest was not present at start of the study** | **Comparability of cohorts based on the design or analysis** | **Assessment of outcome** | **Was follow-up long enough for outcomes to occur** | **Adequacy of follow-up cohorts** | **Quality score** |
| Hayward et al., 2023 | 1 | 1 | 1 | 1 | 2 | 1 | 1 | 1 | 9 |
| Dong J et al., 2021 | 1 | 0 | 1 | 1 | 2 | 1 | 1 | 1 | 8 |
| Kong X et al,2023 | 1 | 1 | 1 | 1 | 2 | 1 | 0 | 1 | 8 |
| Sandokji I et al.,2020 | 1 | 1 | 1 | 1 | 1 | 1 | 1 | 1 | 8 |
| Musial, K et al.,2023 | 1 | 0 | 1 | 1 | 2 | 1 | 1 | 1 | 8 |
| Musial et al., 2024 | 1 | 0 | 1 | 1 | 2 | 1 | 0 | 1 | 8 |
| Luo XQ et al., 2023 | 1 | 1 | 1 | 1 | 2 | 1 | 1 | 1 | 9 |
| Hu J et al., 2024 | 1 | 1 | 1 | 1 | 2 | 1 | 1 | 1 | 9 |
| Zeng X et al., 2022 | 1 | 1 | 1 | 1 | 2 | 1 | 1 | 1 | 9 |
| Nagy M et al., 2024 | 1 | 1 | 1 | 1 | 2 | 1 | 1 | 1 | 9 |
| Fragasso T et al.,2023 | 1 | 1 | 1 | 1 | 2 | 1 | 1 | 1 | 9 |

The Newcastle-Ottawa Scale (NOS) was used to assess the risk of bias (RoB) across the 11 included studies. Observational studies with scores ≥7 points were considered of “good quality,” and studies with 4-6 points were considered “fair quality.” Studies with ≤ 3 points were considered as “poor quality.” The RoB analysis showed that most studies presented a minimal risk in all major domains, including cohort representativeness, exposure ascertainment, and follow-up adequacy. Nonetheless, high or unclear risks were found in specific domains regarding selecting non-exposed cohorts and follow-up adequacy pointing out potential methodological limitations. One study was rated as having a moderate overall risk. The remaining studies were rated as low risk. The assigned weights ranged from 6 to 9 points, representing the level of confidence in the strength of each study's findings. The findings suggest that most studies are of high methodological quality, highlighting their robustness and reliability, supporting contributions to the systematic review.

**Supplemental Figure 1:** a) A traffic light plot of the risk of bias (RoB) assessment using the Newcastle-Ottawa Scale (NOS) was conducted across the 11 included studies, categorizing the risk level for each domain within each study as high, moderate, or low. b) Domain-wise summary of RoB analysis.

**
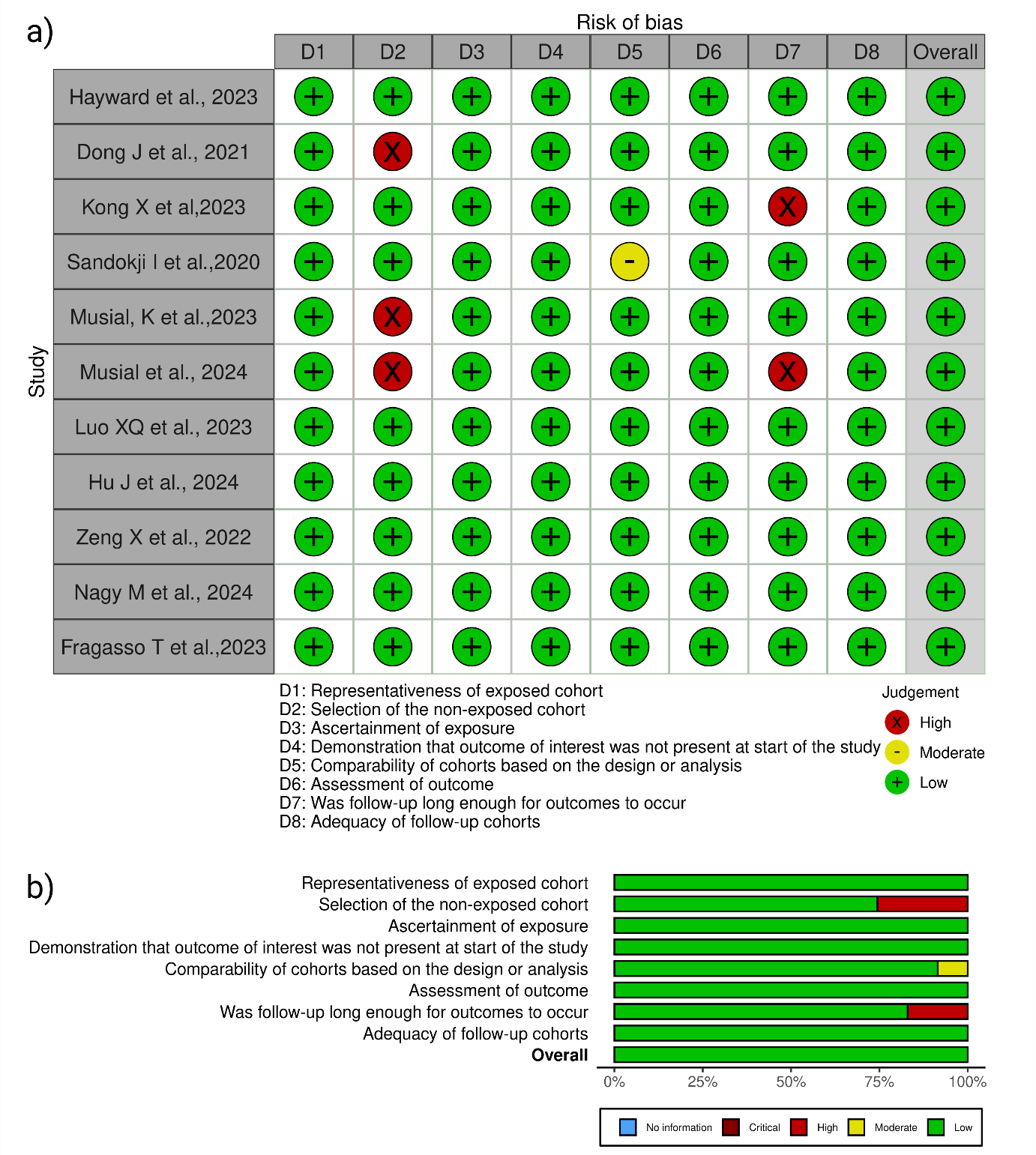
**

**Supplemental Table 2:** Characteristics of the included studies

| **Author, year** | **Study design** | **Single / Multi center** | **Geography** | **Patient population** | **Data source** | **Mean / Median age (years)** | **% AKI (No. of AKI / Sample size)** | **AKI definition** |
| --- | --- | --- | --- | --- | --- | --- | --- | --- |
| Fragasso T et al., 2023 | Retro-spective | single | Italy | Admitted to the PCICU | EHR / EMR | 0.45 | 83.1% (348/419) | KDIGO |
| Nagy M et al., 2024 | Retro-spective | single | USA | Who underwent cardiothoracic surgeries | EHR / EMR | 0.5 | 37%  (30/81) | KDIGO |
| Hu J et al., 2024 | Pro-spective | Multi-center | China | Critically ill children admitted to PICUs | EHR / EMR | 2.67 | 27.4%  (79/288) | KDIGO |
| Musiał et al., 2024 | Retro-spective | single | Poland | undergoing their first allogeneic HSCT | Medical records | 8.27 | 63.2%  (12/19) | pRIFLE |
| Sandokji I et al., 2020 | Retro-spective | Multi-center | USA | Hospitalized children with at least two creatinine values measured during admission | EHR / EMR | 4.4 | 8.9%  (750/8473) | KDIGO |
| Luo XQ et al., 2023 | Retro-spective | Multi-center | China | Undergoing cardiac surgery with cardiopulmonary bypass​ | EHR / EMR | 1 | 15.9%  (615/3863) | KDIGO |
| Kong X et al., 2023 | Retro-spective | single | China | Undergoing aortic arch reconstruction with cardiopulmonary bypass | Retro-spective data | 0.17 | 50%  (67/134) | KDIGO and RIFLE criteria |
| Zeng X et al., 2022 | Retro-spective | single | China | Undergoing congenital heart surgery | Pediatric Intensive Care (PIC) database | 1 | 9.8%  (331/3386) | KDIGO |
| Dong J et al., 2021 | Retro-spective | Multi-center | USA and UK | critical care patients | EHR / EMR | 4 | 11.4%  (1922/16863) | KDIGO |
| Hayward et al., 2023 | Retro-spective | single | UK | cardiac surgery with cardio-pulmonary bypass for congenital heart disease | NICOR validated dataset | 0.58 | 25.8%  (102/396) | KDIGO |
| Musiał K et al., 2023 | Pro-spective | single | Poland | Undergoing first allogeneic HSCT | clinical and laboratory data | 4.5 | 14.8%  (4/27) | pRIFLE |

NICOR: National Institute for Cardiovascular Outcomes Research

**Supplemental Table 3:** Summary of AI/ML Models included in our review

| ***AI/ML Model*** | ***Sub-types*** | ***No. of Studies*** | ***Models used*** |
| --- | --- | --- | --- |
| Logistic Regression | Logistic Regression, Forward Selection Method, LASSO Method | 5 | 11 |
| Random Forest | - | 5 | 5 |
| Gradient Boosting | Gradient Boosting Machine (GBM), Light Gradient Boosting Machine (LGBM), Extreme Gradient Boosting (XGB) | 4 | 10 |
| Support Vector Machine | - | 3 | 4 |
| Neural Network | Artificial Neural Network (ANN), Multilayer Perceptron (MLP), Long Short-Term Memory (LSTM), Gate Recurrent Units (GRU), Reverse Time Attention Model (RETAIN), Time-Aware Attention-Based Recurrent Neural Network (RNN) | 3 | 8 |
| Ensemble Learning | Adaptive Boosting, Age-Dependent Ensemble Machine Learning Model | 2 | 2 |
| Decision Tree | - | 1 | 1 |
| Extra Tree | - | 1 | 1 |
| Genetic Algorithm | - | 1 | 3 |
| Clinical | - | 1 | 3 |
| Top AUC | - | 1 | 3 |
| GaussianNB (GNB) | - | 1 | 2 |
| Dipole | - | 1 | 1 |
| KNN | - | 1 | 1 |

**Supplemental Table 4: Features for different AI models across the studies**

| **Author, year** | **Cohorts** | **AL model** | **AL model category** | **Number of features** | **Feature selection method** | **Top features** |
| --- | --- | --- | --- | --- | --- | --- |
| Fragasso T et al.,2023 | Test | RF | RF | 36 | - | Creatinine, basal creatinine, platelets count, adrenaline support, and lactate dehydrogenase |
| Nagy M et al., 2024 | Test | Light GBM | GBM | 34 | SHAP | Preoperative serum creatinine; surgery duration; POD0 serum pH; POD0 lactate; Cardiopulmonary bypass duration; POD0 vasoactive inotropic score; gender; POD0 hematocrit; Preoperative weight; POD0 serum creatinine |
| Hu J et al., 2024 | Internal Validation | RF | RF | 33 | SHAP | Body weight, Pediatric Risk of Mortality III score, MAP minimum, activated partial thromboplastin time, Total bilirubin, eGFR, blood urea nitrogen, urine output, Lactate, serum creatinine |
| Hu J et al., 2024 | Internal Validation | Light GBM | GBM |  |  |  |
| Hu J et al., 2024 | Internal Validation | GBM | GBM |  |  |  |
| Hu J et al., 2024 | Internal Validation | XGboost | GBM |  |  |  |
| Hu J et al., 2024 | Internal Validation | Adaptive boosting | ELT |  |  |  |
| Hu J et al., 2024 | Internal Validation | SVM | SVM |  |  |  |
| Hu J et al., 2024 | Internal Validation | LR | LR |  |  |  |
| Hu J et al., 2024 | Internal Validation | ANN | NN |  |  |  |
| Musiał et al., 2024 | - | RF | RF | 8 | Feature importance | eGFR before HSCT, eGFR after HSCT, methotrexate use, cytomegalovirus (CMV) infection, adenovirus (ADV) infection, acute GvHD occurrence, mycophenolate mofetil use, glucocorticoid use. |
| Sandokji I et al.,2020 | Test | Lasso | LR | 10 | LASSO | Blood Urea Nitrogen (BUN), Creatinine, Glucose, Time Since Admission, Sodium Bicarbonate Use, Oxygen Saturation, Change in Creatinine Over 48 Hours, Alprostadil Use, Calcium, Calcium Gluconate Use. |
| Sandokji I et al.,2020 | Test | Forward | LR |  | Forward selection |  |
| Sandokji I et al.,2020 | Internal Validation | Lasso | LR |  | LASSO |  |
| Sandokji I et al.,2020 | Internal Validation | Forward | LR |  | Forward selection |  |
| Sandokji I et al.,2020 | External Validation | Lasso | LR |  | LASSO |  |
| Sandokji I et al.,2020 | External Validation | Forward | LR |  | Forward selection |  |
| Luo XQ et al., 2023 | Test | XGBoost | GBM | 27 | Combined 4 methods | Baseline serum creatinine (SCr), perfusion time, body length, operation time, intraoperative blood loss​ |
| Luo XQ et al., 2023 | External Validation | XGBoost | GBM |  |  |  |
| Kong X et al,2023 | Train | LR | LR | 15 | LASSO | Renal ischemia, cyanosis, eGFR, Patent Ductus Arteriosus, weight, newborn, premature, surgery duration, CPB strategy, gender |
| Kong X et al,2023 | Train | Light GBM | GBM |  |  |  |
| Kong X et al,2023 | Train | MLP | NN |  |  |  |
| Kong X et al,2023 | Train | SVM | SVM |  |  |  |
| Kong X et al,2023 | Train | XGboost | GBM |  |  |  |
| Kong X et al,2023 | Test | LR | LR |  |  |  |
| Kong X et al,2023 | Test | Light GBM | GBM |  |  |  |
| Kong X et al,2023 | Test | MLP | NN |  |  |  |
| Kong X et al,2023 | Test | SVM | SVM |  |  |  |
| Kong X et al,2023 | Test | XGBoost | GBM |  |  |  |
| Zeng X et al., 2022 | - | LR | LR | 20 | SHAP | Autologous blood transfusion, mechanical ventilation time, Fresh Frozen Plasma transfusion, C-reactive protein, cardiopulmonary bypass time, aortic cross-clamp time, platelet count, lymphocytes, pressure of oxygen, age |
| Zeng X et al., 2022 | - | SVM | SVM |  |  |  |
| Zeng X et al., 2022 | - | LSTM | NN |  |  |  |
| Zeng X et al., 2022 | - | GRU | NN |  |  |  |
| Zeng X et al., 2022 | - | RETAIN | NN |  |  |  |
| Zeng X et al., 2022 | - | RNN* | NN |  |  |  |
| Dong J et al., 2021 | Train​ | Ensemble machine learning^ | ELT | 15 |  | Creatinine rate of change, Shock index, SpO2, Blood urea nitrogen, Bilirubin​ |
| Hayward et al., 2023 | - | RF | RF | 7 | Random forest variable importance plot and multi-collinearity testing | Age, CPB duration, aortic cross-clamp time, antenatal diagnosis, and oxygen delivery <350 mL/min/m² |
| Hayward et al., 2023 | - | LR | LR |  |  |  |
| Musiał, K et al.,2023 | Train | RF | RF | 3 | Recursive feature selection | KIM-1, IL-18, and NGAL |

RF – Random Forest; GBM – Gradient Boosting; ELT – ensemble learning technique; SVM – Support Vector Machine; NN – Neural Network; LR – Logistic regression; MLP - Multilayer Perceptron; GRU - Rate recurrent units; POD0 – Post-operative day 0; MAP - mean arterial pressure; eGFR - estimated glomerular filtration rate; HSCT - Hematopoietic Stem Cell Transplantation; GvHD - Graft-versus-Host Disease; CPB - Cardiopulmonary Bypass

*Time-aware attention-based RNN (a time-aware RNN to embed time information and a time-aware key-query attention mechanism to identify the key time steps among patients’ historical events in their electronic health record data in the proposed model)

^age-dependent

**Supplementary Table 5:** Meta-analysis of area under the curve (AUC) for three AI/ML models in assessing AKI among pediatric patients stratified by different variables

| AI Model | Variables | | No. of studies, cohorts | No. of AKI / Model sample size | AUC (95% CI) |
| --- | --- | --- | --- | --- | --- |
| Gradient boosting | Study design | Retrospective cohort study | 3 studies, 7 cohorts | 779/4,212 | **0.87 (0.84-0.91)** |
|  | Data source | EHR / EMR | 2 studies, 3 cohorts | 645/3,944 | **0.89 (0.88-0.91)** |
|  |  | Non EHR / EMR | 1 study, 4 cohorts | 134/268 | 0.79 (0.72-0.85) |
|  | Age | <1 year | 2 study, 5 cohorts | 164/349 | 0.80 (0.74-0.86) |
|  |  | >=1 year | 1 study, 2 cohorts | 615/3,863 | **0.90 (0.88-0.92)** |
|  | AKI definition | KDIGO | 2 studies, 3 cohorts | 645/3,944 | **0.89 (0.88-0.91)** |
|  |  | KDIGO and RIFLE criteria | 1 study, 4 cohorts | 134/268 | 0.79 (0.72-0.85) |
| Logistic regression | Study design | Retrospective cohort study | 3 studies, 9 cohorts | 1898/20,466 | 0.79 (0.76-0.82) |
|  | Data source | EHR / EMR | 1 study, 6 cohorts | 1500/16,946 | 0.77 (0.75-0.79) |
|  |  | Non EHR / EMR | 2 studies, 3 cohorts | 398/3,520 | **0.84 (0.77-0.92)** |
|  | Age | <1 year | 1 study, 2 cohorts | 67/134 | **0.87 (0.82-0.91)** |
|  |  | >=1 year | 2 studies, 7 cohorts | 1831/20,332 | 0.78 (0.76-0.79) |
|  | AKI definition | KDIGO | 2 studies, 7 cohorts | 1831/20,332 | 0.78 (0.76-0.79) |
|  |  | KDIGO and RIFLE criteria | 1 study, 2 cohorts | 67/134 | **0.87 (0.82-0.91)** |
| Neural network | Study design | Retrospective cohort study | 2 studies, 6 cohorts | 1,391/13,678 | 0.77 (0.70-0.84) |
|  | Data source | EHR / EMR | 1 study, 2 cohorts | 67/134 | 0.59 (0.56-0.63) |
|  |  | Non EHR / EMR | 1 study, 4 cohorts | 1,324/13,544 | 0.81 (0.77-0.86) |
|  | Age | <1 year | 1 study, 2 cohorts | 67/134 | 0.59 (0.56-0.63) |
|  |  | >=1 year | 1 study, 4 cohorts | 1,324/13,544 | 0.81 (0.77-0.86) |
|  | AKI definition | KDIGO | 1 study, 4 cohorts | 1,324/13,544 | 0.81 (0.77-0.86) |
|  |  | KDIGO and RIFLE criteria | 1 study, 2 cohorts | 67/134 | 0.59 (0.56-0.63) |

**Supplemental Table 6:** Performance metrics for different AI models across the studies

| **Author, year** | **Cohorts** | **AL model** | **AUC** | **Sensitivity** | **Specificity** | **PPV** | **NPV** | **Accuracy** | **F1 score** |
| --- | --- | --- | --- | --- | --- | --- | --- | --- | --- |
| Fragasso T et al.,2023 | Test | RF | 0.93 | 0.71 | 0.98 | 0.92 | 0.92 | - | - |
| Nagy M et al., 2024 | Test | GBM | 0.88 | 0.63 | - | 0.92 | - | 0.91 | 0.73 |
| Hu J et al., 2024 | Internal Validation | RF | 0.94 | 0.873 | 0.904 | 0.775 | 0.95 | 0.896 | 0.821 |
| Hu J et al., 2024 | Internal Validation | GBM | 0.936 | 0.899 | 0.876 | 0.732 | 0.958 | 0.882 | 0.807 |
| Hu J et al., 2024 | Internal Validation | GBM | 0.922 | 0.873 | 0.866 | 0.711 | 0.948 | 0.868 | 0.784 |
| Hu J et al., 2024 | Internal Validation | GBM | 0.92 | 0.81 | 0.919 | 0.79 | 0.928 | 0.889 | 0.8 |
| Hu J et al., 2024 | Internal Validation | ELT | 0.918 | 0.835 | 0.9 | 0.759 | 0.935 | 0.882 | 0.795 |
| Hu J et al., 2024 | Internal Validation | SVM | 0.84 | 0.747 | 0.799 | 0.584 | 0.893 | 0.785 | 0.656 |
| Hu J et al., 2024 | Internal Validation | LR | 0.829 | 0.696 | 0.837 | 0.618 | 0.879 | 0.799 | 0.655 |
| Hu J et al., 2024 | Internal Validation | NN | 0.792 | 0.671 | 0.88 | 0.679 | 0.876 | 0.823 | 0.675 |
| Musiał et al., 2024 | - | RF | 0.839 | 0.842 | - | 0.852 | - | 0.842 | - |
| Sandokji I et al.,2020 | Test | LR | 0.77 | - | - | - | - | - | - |
| Sandokji I et al.,2020 | Test | LR | 0.78 | - | - | - | - | - | - |
| Sandokji I et al.,2020 | Internal Validation | LR | 0.73 | - | - | - | - | - | - |
| Sandokji I et al.,2020 | Internal Validation | LR | 0.73 | - | - | - | - | - | - |
| Sandokji I et al.,2020 | External Validation | LR | 0.79 | - | - | - | - | - | - |
| Sandokji I et al.,2020 | External Validation | LR | 0.84 | - | - | - | - | - | - |
| Luo XQ et al., 2023 | Test | GBM | 0.912 | 0.951 | 0.583 | 0.322 | 0.983 | - | - |
| Luo XQ et al., 2023 | External Validation | GBM | 0.889 | 0.804 | 0.805 | 0.283 | 0.977 | - | - |
| Kong X et al,2023 | Train | LR | 0.889 | 0.824 | 0.807 | 0.86 | 0.841 | 0.547 | 0.813 |
| Kong X et al,2023 | Train | GBM | 0.797 | 0.661 | 0.721 | 0.735 | 0.775 | 0.495 | 0.618 |
| Kong X et al,2023 | Train | NN | 0.582 | 0.644 | 0.711 | 0.59 | 0.636 | 0.477 | 0.69 |
| Kong X et al,2023 | Train | SVM | 0.731 | 0.679 | 0.877 | 0.513 | 0.619 | 0.406 | 0.8 |
| Kong X et al,2023 | Train | GB | 0.878 | 0.812 | 0.836 | 0.821 | 0.839 | 0.501 | 0.792 |
| Kong X et al,2023 | Test | LR | 0.84 | 0.792 | 0.795 | 0.851 | 0.796 | 0.547 | 0.778 |
| Kong X et al,2023 | Test | GB | 0.717 | 0.584 | 0.839 | 0.555 | N/A | 0.495 | 0.574 |
| Kong X et al,2023 | Test | NN | 0.619 | 0.601 | 0.767 | 0.611 | 0.66 | 0.477 | 0.66 |
| Kong X et al,2023 | Test | SVM | 0.702 | 0.681 | 0.777 | 0.716 | 0.712 | 0.501 | 0.689 |
| Kong X et al,2023 | Test | GB | 0.743 | 0.812 | 0.836 | 0.821 | 0.839 | 0.501 | 0.792 |
| Zeng X et al., 2022 | - | LR | 0.795 | 0.706 | - | - | - | 0.731 | - |
| Zeng X et al., 2022 | - | SVM | 0.817 | 0.79 | - | - | - | 0.759 | - |
| Zeng X et al., 2022 | - | NN | 0.775 | 0.708 | - | - | - | 0.719 | - |
| Zeng X et al., 2022 | - | NN | 0.806 | 0.713 | - | - | - | 0.737 | - |
| Zeng X et al., 2022 | - | NN | 0.799 | 0.705 | - | - | - | 0.73 | - |
| Zeng X et al., 2022 | - | NN | 0.874 | 0.813 | - | - | - | 0.798 | - |
| Dong J et al., 2021 | Train​ | ELT | 0.75 | 0.27 | 0.96 | 0.44 | 0.91 | - | - |
| Hayward et al., 2023 | - | RF | 0.671 | - | - | - | - | 0.66 | - |
| Hayward et al., 2023 | - | LR | 0.601 | - | - | - | - | 0.65 | - |
| Musiał, K et al.,2023 | Train | RF | 0.833 | 0.800 | - | - | - | - | - |

**Supplemental Table 7:** Pooled analysis of various performance metrics of different machine learning / AI models in assessing AKI among pediatric patients.

| **a) Pooled analysis of sensitivity** | | | | |
| --- | --- | --- | --- | --- |
| Model | No. of studies | No. of cohorts | AKI (n) /  Study sample size (N) | Median (IQR) |
| Ensemble learning technique | 2 | 2 | 2001/17,151 | 0.553 (0.411 - 0.694) |
| Gradient boosting | 4 | 10 | 791/4,366 | 0.810 (0.661 - 0.812) |
| Logistic Regression | 3 | 4 | 477/3,808 | 0.749 (0.704 - 0.801) |
| Neural network | 3 | 7 | 477/3,808 | 0.705 (0.658 - 0.711) |
| Random Forest | 4 | 4 | 443/753 | 0.821 (0.778 - 0.850) |
| Support Vector Machine | 3 | 4 | 477/3,808 | 0.714 (0.680 - 0.758) |
| **b) Pooled analysis of specificity** | | | | |
| Model | No. of studies | No. of cohorts | AKI (n) /  Study sample size (N) | Median (IQR) |
| Ensemble learning technique | 2 | 2 | 2,001/17,151 | 0.930 (0.915 - 0.945) |
| Gradient boosting | 3 | 9 | 761/4,285 | 0.838 (0.828 - 0.869) |
| Logistic Regression | 2 | 3 | 146/422 | 0.807 (0.801 - 0.822) |
| Neural network | 2 | 3 | 146/422 | 0.767 (0.739 - 0.824) |
| Random Forest | 2 | 2 | 427/707 | 0.942 (0.923 - 0.961) |
| Support Vector Machine | 2 | 3 | 146/422 | 0.799 (0.788 - 0.838) |
| **c) Pooled analysis of PPV** | | | | |
| Model | No. of studies | No. of cohorts | AKI (n) /  Study sample size (N) | Median (IQR) |
| ensemble learning technique | 2 | 2 | 2,001/17,151 | 0.600 (0.520 - 0.679) |
| Gradient boosting | 4 | 10 | 791/4366 | 0.735 (0.711 - 0.821) |
| Logistic Regression | 2 | 3 | 146/422 | 0.851 (0.735 - 0.856) |
| Neural network | 2 | 3 | 146/422 | 0.611 (0.601 - 0.645) |
| Random Forest | 4 | 4 | 443/753 | 0.860 (0.833 - 0.880) |
| Support Vector Machine | 2 | 3 | 146/422 | 0.584 (0.549 - 0.650) |
| **d) Pooled analysis of NPV** | | | | |
| Model | No. of studies | No. of cohorts | AKI (n) /  Study sample size (N) | Median (IQR) |
| ensemble learning technique | 2 | 2 | 2,001/17,151 | 0.923 (0.916 - 0.929) |
| Gradient boosting | 3 | 8 | 761/4285 | 0.934 (0.839 - 0.963) |
| Logistic Regression | 2 | 3 | 146/422 | 0.841 (0.819 - 0.860) |
| Neural network | 2 | 3 | 146/422 | 0.660 (0.648 - 0.768) |
| Random Forest | 2 | 2 | 427/707 | 0.935 (0.928 - 0.943) |
| Support Vector Machine | 2 | 3 | 146/422 | 0.712 (0.666 - 0.803) |
| **e) Pooled analysis of accuracy** | | | | |
| Model | No. of studies | No. of cohorts | AKI (n) /  Study sample size (N) | Median (IQR) |
| ensemble learning technique | 1 | 1 | 79/288 | - |
| Gradient boosting | 3 | 8 | 176/503 | 0.685 (0.501 - 0.884) |
| Logistic Regression | 4 | 5 | 579/4,204 | 0.650 (0.547 - 0.731) |
| Neural network | 3 | 7 | 477/3,808 | 0.730 (0.598 - 0.768) |
| Random Forest | 4 | 4 | 197/730 | 0.821 (0.765 - 0.856) |
| Support Vector Machine | 3 | 4 | 477/3,808 | 0.630 (0.477 - 0.766) |
| **f) Pooled analysis of F1 score** | | | | |
| Model | No. of studies | No. of cohorts | AKI (n) /  Study sample size (N) | Median (IQR) |
| ensemble learning technique | 1 | 1 | 79/288 | - |
| Gradient boosting | 3 | 8 | 176/503 | 0.788 (0.702 - 0.794) |
| Logistic Regression | 2 | 3 | 146/422 | 0.778 (0.717 - 0.796) |
| Neural network | 2 | 3 | 146/422 | 0.675 (0.668 - 0.683) |
| Random Forest | 1 | 1 | 79/288 | - |
| Support Vector Machine | 2 | 3 | 146/422 | 0.689 (0.673 - 0.745) |
